# Predictability of Arctic sea ice on weather time scales

**DOI:** 10.1038/s41598-018-24660-0

**Published:** 2018-04-25

**Authors:** M. Mohammadi-Aragh, H. F. Goessling, M. Losch, N. Hutter, T. Jung

**Affiliations:** 1 Alfred Wegener Institute, Helmholtz Centre for Polar and Marine Research, Bremerhaven, Germany; 2 University of Bremen, Bremen, Germany; 3 Present Address: Institute of Coastal Research, Helmholtz-Zentrum Geesthacht, Geesthacht, Germany

## Abstract

The field of Arctic sea ice prediction on “weather time scales” is still in its infancy with little existing understanding of the limits of predictability. This is especially true for sea ice deformation along so-called Linear Kinematic Features (LKFs) including leads that are relevant for marine operations. Here the potential predictability of the sea ice pack in the wintertime Arctic up to ten days ahead is determined, exploiting the fact that sea ice-ocean models start to show skill at representing sea ice deformation at high spatial resolutions. Results are based on ensemble simulations with a high-resolution sea ice-ocean model driven by atmospheric ensemble forecasts. The predictability of LKFs as measured by different metrics drops quickly, with predictability being almost completely lost after 4–8 days. In contrast, quantities such as sea ice concentration or the location of the ice edge retain high levels of predictability throughout the full 10-day forecast period. It is argued that the rapid error growth for LKFs is mainly due to the chaotic behaviour of the atmosphere associated with the low predictability of near surface wind divergence and vorticity; initial condition uncertainty for ice thickness is found to be of minor importance as long as LKFs are initialized at the right locations.

## Introduction

The rapid decline of Arctic sea ice has sparked considerable interest in predicting sea ice due to the projected increase in Arctic maritime operations^1^. As for traditional weather prediction^2^, skilful sea ice forecasts have the potential of saving lives, support emergency management, prevent economic losses from hazardous environmental conditions; and they may also create substantial financial revenue. Such forecasts are also needed to optimize shipping routes, which could be relevant for minimizing pollution.

Recent years have seen substantial improvements in our understanding of Arctic sea ice predictability on seasonal time scales^3–8^. Outcomes from the Sea Ice Outlook, launched in 2008 as an open forum for predicting September Arctic sea ice extent^9^, are a prominent example of the progress made. For marine operations, sea ice forecasts on shorter, daily (“weather”) time scales are also critical, especially when it comes to preparation and decision making on the risk of exceptional situations and for operational decision making. Very little is known, however, when it comes to short-term predictability of Arctic sea ice^10,11^, one reason being that previous predictability research was focussing mostly on the atmosphere and non-polar regions^1^.

When it comes to sea ice, past predictability studies have considered mostly the pan-Arctic sea ice extent, or the more user-relevant location of the ice edge^4^. However, sea ice deformation is of critical importance for decision-making in marine operations within the ice pack because deformation is related to sea ice pressure and leads. The initialization and short-term forecasting of deformation features has thus been identified as a promising as well as challenging research task^12^. Until recently, it was believed that classical sea ice models, employing viscous-plastic rheologies, would not be able to represent deformation-related aspects such as leads which localize along so-called Linear Kinematic Features (LKFs). Here, we determine the limits of predictability of Arctic sea ice on “weather time scales” (i.e. from 1–10 days), exploiting the fact that sea ice-ocean models at very high horizontal resolutions are actually capable of representing observed aspects related to sea ice deformation^1,13–15^. Furthermore, we compare outcomes for sea ice parameters traditionally considered, such as sea ice concentration, with the predictability of LKFs. Given the challenges that come with verifying LKFs due to their sharp and elongated structures, we also consider various verification methods that differ in the degree to which small errors in predicting LKF location are penalized.

This paper is organized as follows: The methods, including the experimental setup, the detection of LKF and the determination of potential predictability are described in Section 2. This is followed by a presentation of the results and a discussion and conclusions section.

## Methods

### Experiments

Our study is based on simulations with the Massachusetts Institute of Technology general circulation model (MITgcm)^16,17^ with a viscous-plastic sea ice component^18^. We use a standard viscous-plastic rheology^19^ in our simulations that relates the sea ice strain rates, concentration, and thickness to the internal stresses. The ice strength depends linearly on the ice thickness and exponentially on ice concentration and consequently the sea ice state is a controlling factor in the generation of LKFs. We use an Arctic-wide grid at a horizontal resolution of ∼4.5 km with 50 vertical layers in the ocean component, spanning one face of the cubed sphere, centered on the North Pole. The open boundaries in the Pacific and the Atlantic are prescribed using monthly mean data taken from a global simulation^20^. Atmospheric forcing fields are taken from either the ERA-Interim Reanalysis^21^ or from individual members of the ECMWF Ensemble Prediction System (EPS)^22^ as detailed below.

**Figure 1:**
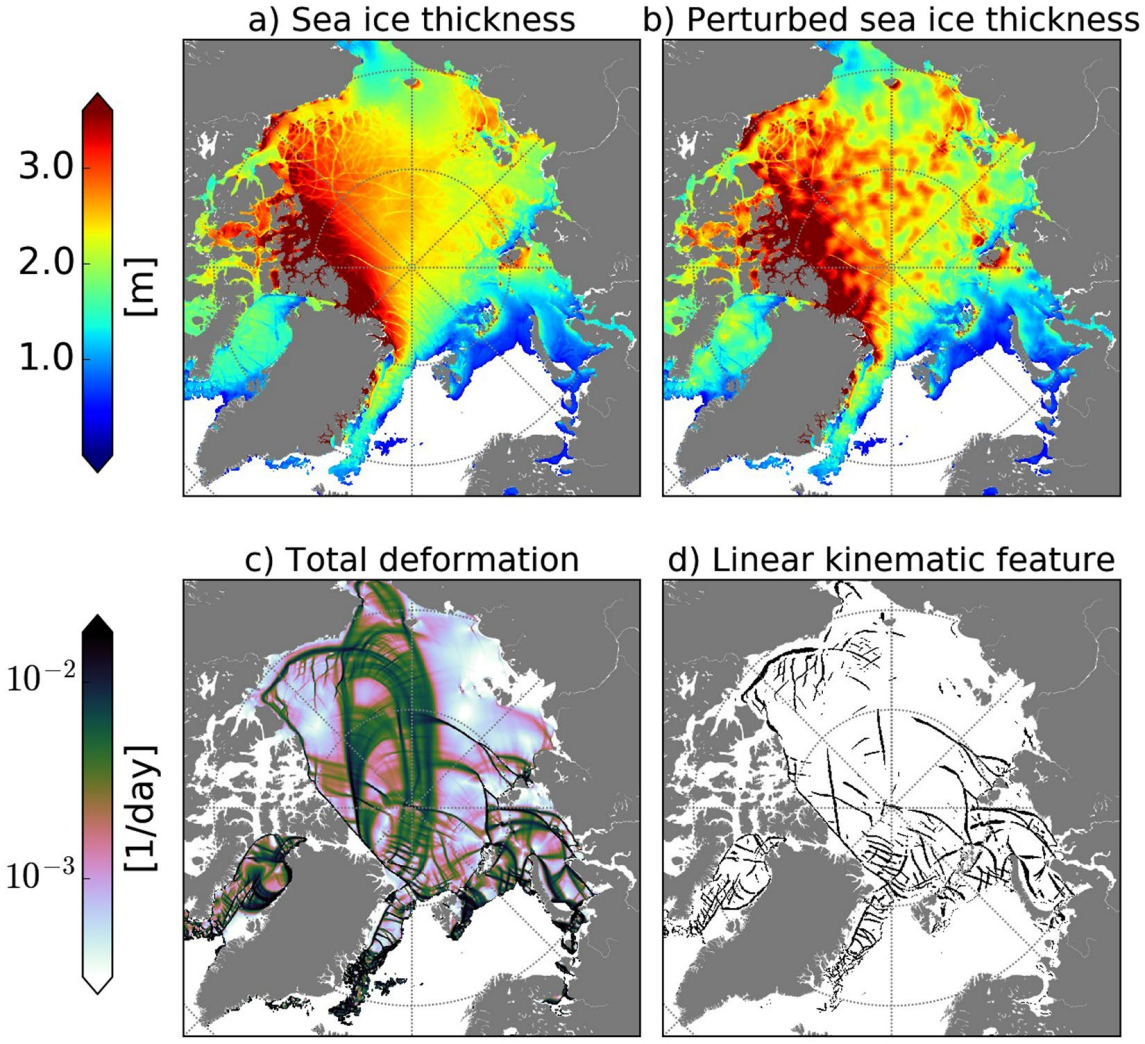
Sea ice thickness initial conditions (in m) on 1 February 2005 with (**a**) atmospheric perturbations only (AtmU) and (**b**) additional sea ice perturbations superimposed (AtmU + IcU). Also shown for AtmU: (**c**) sea ice deformation (in 1/day) and (**d**) the binary map of Linear Kinematic Features (LKFs) associated with (c). All results are based on six-hourly averaged fields. This figure was generated using “Intel Python from Intel Parallel Studio XE Cluster Edition for Linux 2017 Initial Release” that includes “Matplotlib-1.5.1” (https://software.intel.com/en-us/distribution-for-python”) and Basemap-1.0.7 (http://matplotlib.org/basemap/).

A control simulation from 2001 to 2015 forced with ERA-Interim reanalysis data provides initial conditions for all ensemble prediction experiments. Two sets of 15-member ensemble prediction experiments are considered: In the first set (AtmU hereafter), we account only for atmospheric forcing uncertainty associated with the chaotic atmospheric nature: Different sea ice-ocean model realizations are driven with different members of the ECMWF Ensemble Prediction System using the same initial conditions for the sea ice-ocean system. In the second set (AtmU + IcU hereafter), we additionally take into account the uncertainty of the initial conditions for sea ice. This is achieved by perturbing the initial sea ice thickness fields $h$ as follows:

1 $$h^{\prime} =h\cdot \exp (f\cdot \varepsilon )\,,$$

where $h^{\prime}$ is the perturbed sea ice thickness; $f$ is a perturbation strength parameter; and $\varepsilon$ is noise with zero mean and unit variance, derived from white noise that was spatially smoothed with a Gaussian filter with 50 km standard deviation. We choose $f=0.1$ such that the resulting perturbations in Arctic sea ice thickness amount to a maximum of about 0.5 m (see Fig. 1a,b for an example of the perturbed field for a particular day)—an estimate which is consistent with the observational uncertainty for sea ice thickness^23^. Note that by perturbing thickness only, we leave the initial locations of LKFs essentially unchanged.

To reduce the impact of sampling uncertainty, each of the two ensemble experiments was conducted for six different cases by initializing forecasts on the first and fifteenth of the months February, March, and April in 2005. We focus our analysis on February, March and April, when the LKF density distributions are relatively homogenous, noting that LKF predictability might be different in other seasons. We focus on the months around the maximum ice extent because (i) a continuum sea ice model with a 4.5-km grid spacing is thought to best represent a dense ice pack with large floes, (ii) a larger area exhibits LKFs in winter and spring compared to summer and autumn, and (iii) this avoids difficulties in terms of interpretation that might arise from the seasonal cycle in LKF characteristics. The year 2005 was chosen because at that time the operational EPS was technically very similar to the ERA-Interim reanalysis system. Note that for each of the configurations and each of the starting dates, fifteen ensemble members were integrated for ten days (i.e. 10-day forecasts).

We follow a “perfect-model” approach in the sense that we do not quantify forecast errors with respect to actual observations, but study how differences between individual ensemble members evolve, with one of the ensemble members being considered the “truth”. Our analysis thus provides an estimate of *potential* predictability, which typically is regarded as an upper bound for the skill that can be achieved in real operational forecasts^4^.

### LKF detection

Our analysis requires an algorithm that diagnoses linear kinematic features (LKFs) from model output. It is well established to derive LKFs from sea ice deformation fields. The LKFs, indentified in this way, are highly correlated with the spatial pattens of leads^24^. Sea ice shear and divergence also correspond to leads^25^. Various methods have been proposed to detect geomorphological features in sea ice over the past two decades, but there is no unique detection algorithm^26–29^. The most straightforward approach is to use a fixed threshold for distinguishing linear (kinematic) features from background fields^24,26,27,30^. Obviously, the result depends on the threshold value. Given that zones of shear as well as convergence and divergence of sea ice are concentrated along LKFs, we opt for a simple method that is based solely on the total deformation rate of sea ice: Regions with sea ice concentration below 80% are excluded from the analysis. After taking the logarithm of total deformation, we subtract the local background which is determined by applying a 2D Gaussian filter with a standard deviation of five grid cells (∼20 km). Grid cells are considered to belong to an LKF if total deformation exceeds the background deformation by at least a factor of ${10}^{0.2}\approx 1.6$ (Fig. 1d). Note that this approach takes into account that atmospheric winds and coastal geometry impose spatial variations in background deformation of the sea ice. The character of sea ice deformation and the diagnosed LKFs can be inferred from Fig. 1c and d for 1 February 2005.

### Measuring potential predictability

To quantify the difference between pairs of ensemble members (i.e. “forecast error”), we use the simple spatial Pearson correlation as well as the more sophisticated Modified Hausdorff Distance [MHD^31^]. Applied to a pair of LKF patterns, the MHD determines the average distance between any LKF grid cell to the closest such grid cell in the other pattern. In contrast to the spatial correlation, which strongly penalizes even small errors in the predicted location of the LKFs, the MHD can discriminate between small and large differences in LKF location. Given that MHD is constrained to binary fields, we use it to determine the predictability of LKFs only. In contrast, spatial correlations are determined for LKFs as well as several continuous fields including sea ice total deformation anomaly, sea ice concentration anomaly, and the divergence of near-surface winds.

To determine potential predictability PP, we relate the above metrics, averaged over all possible pairs of ensemble members and cases, to their climatological saturation levels. For the spatial correlation this can be expressed as follows:

2 $${{\rm{PP}}}_{\rho }=1-\frac{1-{\rho }_{E}}{1-{\rho }_{C}},$$

where ${\rho }_{E}$ denotes the mean spatial correlation between ensemble members, and ${\rho }_{C}$ is the climatological spatial correlation, that is, the spatial correlation between pairs of states from the same time of the year but taken from different years of the control run, averaged over all possible pairs of the years 2001 to 2015 from February to April. Similarly, potential predictability for MHD is determined as follows:

3 $${{\rm{PP}}}_{{\rm{MHD}}}=1-\frac{{{\rm{MHD}}}_{E}}{{{\rm{MHD}}}_{C}}\,\mathrm{.}$$

Equations (2) and (3) are consistent with skill scores used elsewhere by the forecast verification community^32^

### Data Availability

The datasets generated during the current study are available from the corresponding author on request.

## Results

**Figure 2:**
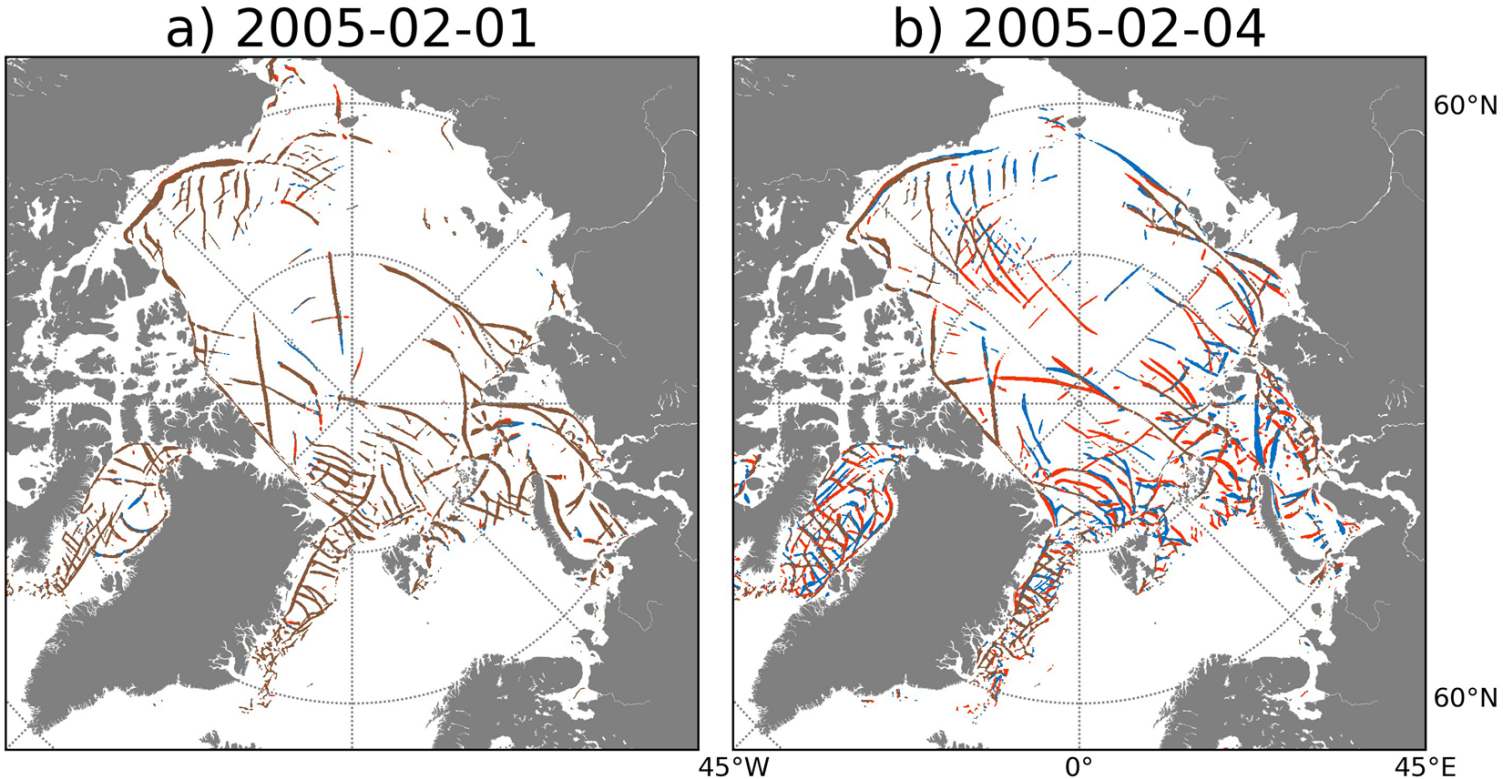
Predictions of Linear Kinematic Features (LKFs) at lead times of (**a**) 1 day and (**b**) 4 days for two randomly chosen ensemble members with atmospheric perturbations only (AtmU). Both forecasts have been initialized on 1 February 2005 at 0 UTC. The LKFs of the two forecasts are marked in red and blue. Where they overlap they are gray, hence gray LKFs indicate agreement between the two forecasts in terms of the predicted location of the LKFs; in contrast, red (first member) and blue (second member) LKFs indicate mismatch between the two forecasts. For 1-day (4-day) forecasts the correlation between the LKFs amounts to 0.9 (0.5); for the Modified Hausdorff Distance (MHD) values of 5.6 km (16.4 km) are obtained. This figure was generated using “Intel Python from Intel Parallel Studio XE Cluster Edition for Linux 2017 Initial Release” that includes “Matplotlib-1.5.1” (https://software.intel.com/en-us/distribution-for-python”) and Basemap-1.0.7 (http://matplotlib.org/basemap/).

To provide a more intuitive insight into the predictability of LKFs, we first consider an individual forecast case. Figure 2 shows 1-day and 4-day predictions of LKFs, initialized on 1 February 2005, from two ensemble members with atmospheric uncertainty only (AtmU). There is evidence for relatively high potential predictability of LKFs during the first day of the forecast (Fig. 2a). In weather prediction this time range spans what is usually referred to as nowcasting and early short-range forecasting. Visual inspection of Fig. 2b reveals a much larger difference between the two ensemble members 4 days into the forecast, suggesting that the potential predictability in the early medium-range, at least for some of the simulated LKFs, is already quite small.

**Figure 3:**
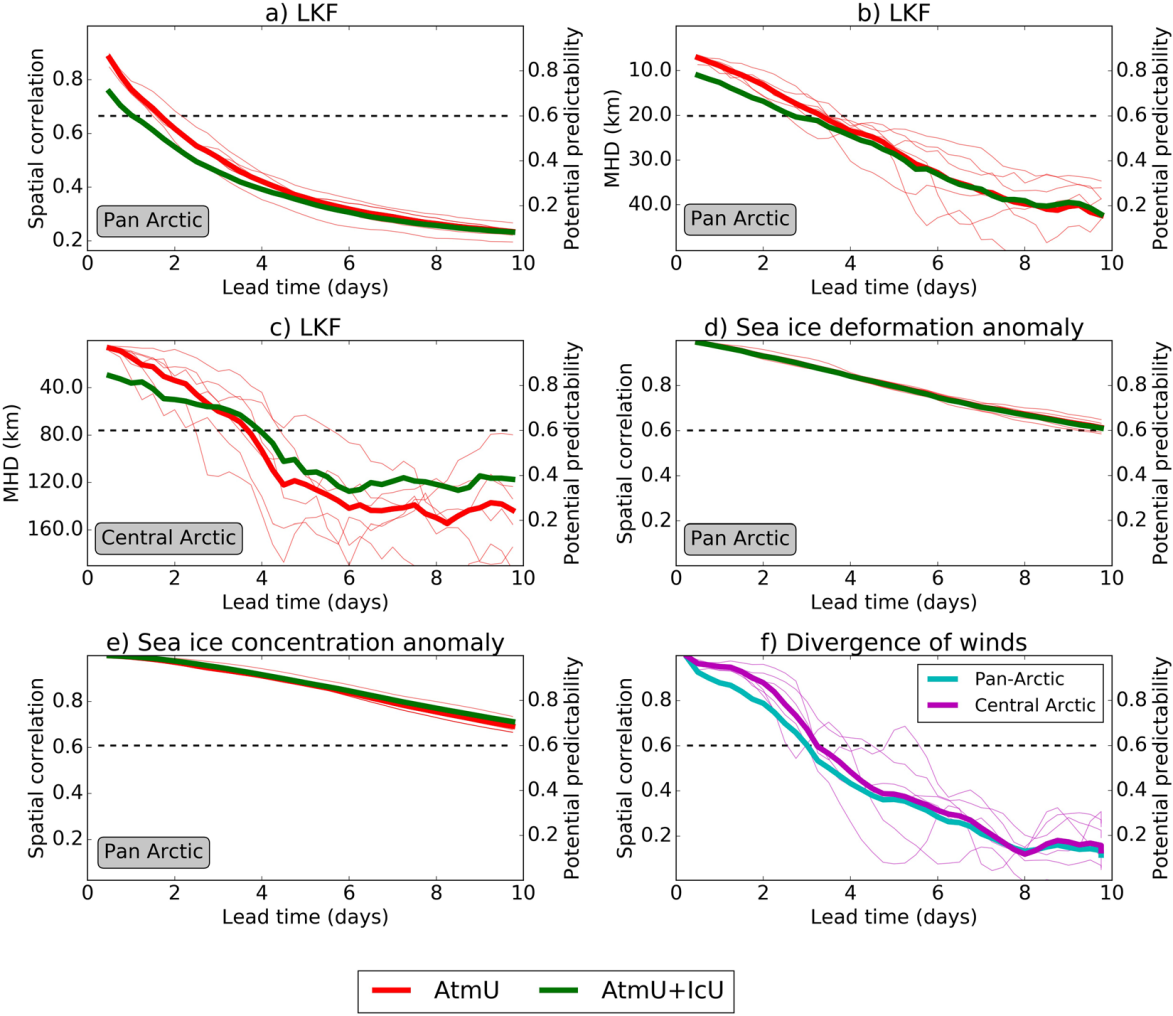
(**a**) Spatial correlation coefficient (left axis) and potential predictability (right axis) for LKFs of pan-Arctic sea ice as a function of forecast lead time for the ensemble experiment with atmospheric uncertainty only (AtmU, red curve) and the one with atmospheric and initial sea ice uncertainty combined (AtmU + IcU, green curve). (**b**) as in (a), but for Modified Hausdorff Distance (MHD in km) and corresponding potential predictability. (**c**) as in (b), but for sea ice in the central Arctic only (80–90°N, 120°E–240°E) (**d**) as in (a), but for sea ice deformation anomalies. (**e**) as in (a), but for sea ice concentration anomalies. (**f**) as in (a), but for the divergence of near-surface winds with atmospheric forcing only and for the whole Arctic (blue) as well as the central Arctic (purple). The dashed gray lines mark a common threshold of useful potential predictability for deterministic metrics^43^. Results are based on all 6 cases (initial times) and 15 ensemble members. The thin curves represent the ensemble means for each individual case.

More quantitative estimates of predictability are provided by combining all ensemble experiments for the 6 different initialization times (Fig. 3). From the average spatial correlation coefficients for the pan-Arctic area (north of 54°N) a rapid drop in skill with increasing lead time can be inferred (Fig. 3a), with correlation coefficients dropping below 0.6 (i.e. less than 36% of the variance explained) by day 2; by day 4 the correlation has dropped further to about 0.4. A comparison between the experiment with atmospheric forcing uncertainty only (AtmU) and with both atmospheric and initial sea ice uncertainty (AtmU-IcU) shows that it is primarily atmospheric uncertainty that is responsible for the rapid error growth. This is especially true for lead times beyond 2 days or so.

Comparing LKF forecasts with a metric like the spatial correlation coefficient, which is widely used in forecast verification, has its limitation, because LKF forecasts represent binary information (either there is an LKF or not), and all location errors, whether small or large, are penalized alike. It is therefore useful to also consider a metric such as the MHD that is better suited for binary information and that can discriminate between small and large displacement errors. The MHD for all forecast cases along with the corresponding potential predictability is shown in Fig. 3b for the pan-Arctic region. Evidently, the MHD-based potential predictability of LKFs remains above 0.6 for longer than that based on the spatial correlation coefficients. Even for longer lead times up to 10 days the MHD metric indicates some potential skill in predicting LKFs. As for the spatial correlation coefficient, atmospheric uncertainty dominates over initial sea ice uncertainty, especially beyond 3 days into the forecast.

So far, the discussion has focussed on the entire Arctic. However, it could be argued that the nature and hence predictability of LKFs in the interior of the Arctic, where relatively thick ice prevails and the effect of surrounding coastlines decreases, is different from regions near the sea ice margins. Figure 3c shows the MHD and corresponding potential predictability for the central Arctic, which here is simply defined as being north of 80° N and between 120°E–240°E. It turns out that the potential predictability in the central Arctic is slightly larger than for the pan-Arctic region, which we attribute to the presence of thicker, hence stronger and less mobile sea ice. At the same time, however, the difference between the different ensemble experiments (thin lines in Fig. 3) is larger and noisier. One explanation for this is that fewer LKFs are found in the interior of the Arctic (Fig. 1) compared to the sea ice margins [e.g.^13^, their Fig. 2], so that the metrics are subject to more sampling variability; thus a larger number of cases are required to obtain more robust statistics.

It is informative to put the predictability of LKFs into the context of that of other variables. The potential predictability for (logarithmized) pan-Arctic sea ice deformation anomalies as determined by means of spatial correlation can be inferred from Fig. 3d. Here *anomalies* instead of full fields were considered in order to avoid that the model’s ability, to simulate climatological patterns of sea ice deformation, contributes to the correlation (by analogy with the widely used 500 hPa geopotential height anomaly correlation). Evidently, the sea ice deformation anomalies are much more predictable than LKFs, with potential predictability going down to 0.6 by day 10 only. This is consistent with the fact that the full sea ice deformation fields include larger spatial scales compared to LKFs (Fig. 1c and d). Note in this context that LKFs are obtained from sea ice deformation by effectively applying a spatial high-pass filter. Interestingly, when the full deformation fields are considered instead of LKFs, initial sea ice perturbations affect the predictions compared to atmospheric uncertainty even less. This suggests that the prediction of larger-scale sea ice aspects is relatively insensitive to perturbations to the initial sea ice thickness at the considered time scale. For anomalies in sea ice concentration very similar results are found (Fig. 3e) with prospects for skilful forecasts up to 10 days and beyond.

To provide an additional point of reference, we computed the pairwise Integrated Ice Edge Error [IIEE^4^] for our ensembles. Averaged over the experiments AtmU and AtmU + IcU and normalised with the climatological IIEE, the corresponding predictability after 10 days is 0.72, which is marginally higher than the predictability of sea-ice concentration anomalies of 0.69 (Fig. 3e).

Atmospheric winds are the most important driver of sea ice changes on daily to subseasonal time scales. It is instructive, therefore, to compare the predictability of sea ice with that of the atmospheric flow. The potential predictiability of the near surface wind divergence, a parameter that is of particular relevance for the generation of leads, shows that the skill for the atmosphere and LKFs is comparable up to about 4 days into the future (Fig. 3f). Thereafter, the predictability of LKFs is larger than that of the divergent wind, suggesting that sea ice provides additional memory that translates into predictability. In general, however, a comparison of atmospheric and sea ice predictability is difficult to carry out due to the strong dependence on the exact variable, metric, and spatial scale being considered [Fig. 3a,d and e;^33,34^].

## Discussion and Conclusions

Experiments with a high-resolution sea ice-ocean model, forced with weather forecasts from the ECWMF ensemble prediction system, have been used to determine the potential predictability of Arctic sea ice on “weather time scales” (1–10 day forecasts), a time range for which skilful forecasts are of critical importance for marine stakeholders in tactical decision making. Our study augments previous work, most of which focussed on longer-term seasonal and interannual prediction^3^. Our results highlight that the predictability of Arctic sea ice on short time scales is critically dependent on the variable being considered. Sea ice concentration anomalies, for example, show high levels of predictability throughout the whole forecast range considered in this study, whereas linear kinematic features (LKFs) such as leads are much less well predictable, especially beyond the first 2–4 days of the forecast. In addition, a skillful prediction of actual LKFs is a challenging task that requires a sophisticated forecasting system with new data assimilation capabilities to obtain accurate initial conditions for sea-ice failure^12^.

Our results suggest that atmospheric uncertainty is more important in generating sea ice forecast error than initial sea ice uncertainty. In this context, we have conducted additional experiments in which we varied the parameters of the initial perturbations. From these experiments we conclude that smaller-scale perturbations introduce spurious additional LKFs, and that the chosen set of parameters appears appropriate. Still, it cannot be excluded that our initial sea ice perturbations are sub-optimal, in the way they reflect real uncertainties and in the way they project onto possible directions of growing forecast error. However, our results are consistent with the notion that sea ice dynamics are primarily determined by atmospheric winds. The higher predictability of wind divergence in the central Arctic (Fig. 3f) might also contribute to higher predictability of sea ice in the central Arctic.

Our study shows that the verification of forecasts of small-scale sea ice features (including determining exact limits of predictability) poses challenges. Traditional measures such as the anomaly correlation coefficient, for example, are quite sensitive to small errors in location. Hence, other less well-established methods such as Modified Hausdorff Distance need to be exploited as well when it comes to user-relevant forecast verification. Future work on sea ice verification can benefit from the expertise gained from the verification of other sharp features such as atmospheric fronts^35^. Furthermore, estimates of deterministic and probabilistic predictability may differ. Therefore, deterministic verification of forecasts, as done in this study essentially by comparing pairs of ensemble members, should be augmented by probabilistic verification where ensemble-derived probabilities are taken into account.

Individual LKFs may not be the only important predictands for shipping or marine operations. Integral properties such as ridge and lead density may also be relevant and probably easier to predict but would require a different type of analysis and potentially different model parameterizations. For example, a ridge density parameterization^36^ or an ice thickness distribution^37,38^ open new fields of application in the context of predictability. With our current model configuration, these questions cannot be addressed.

Simulated small-scale sea ice features depend heavily on the rheology used for the representation of sea ice dynamics. It seems natural, therefore, to ask how model dependent the results from this potential predictability study are. The high intermittency of sea ice deformation found in temporal scaling analyses of drift observations could not be reproduced with viscous-plastic sea ice models at coarse grid resolutions^39^ and only partly for models at higher grid resolution like the one used here^15^. In this light, the potential predictability reported here may be actually slightly overestimated. Repeating our experiments with a new generation of rheologies that produce more intermittency in models^40–42^ may provide enhanced estimates of Arctic sea ice predictability on weather time scales.
